# Mr. Hill's Surgical Remarks

**Published:** 1811-06

**Authors:** George Nesse Hill

**Affiliations:** Chester


### *To the Editors of the Medical and Physical Journal.*

"In cases of wounds attended with denuded pericranium, do not hastily act upon the principle that an exfoliation must sooner or later follow, for this may not eventually be the case one time in ten." Sir William Blizard's M. S. Lectures on Surgery.

GENTLEMEN,

NOTWITHSTANDING the great advances made in surgical practice towards that perfection which it is the honourable aim of the practitioners of the present day to exert every effort to approach, still there exist too many discouraging obstacles to its perfect attainment, without reviving those which increasing knowledge and the result of attentive experiments have happily removed. No. 144 of your Journal contains at p. 146 the following remark, under the title "Theoretical Suggestions for the improvement of Practical Surgery."

"In the operation of trepanning the skull, when the scalp is sufficiently removed it is essential to remove just so much of the pericranium and no more, as the head of the trephine will include, *because the cranium when denuded of its pericranium will, like other bones denuded of their periosteum, grow carious.*" The former part of this quotation, doubtless, contains most useful advice, but I conceive not a new suggestion; no dexterity can justify a wanton use of the knife, nor should an occasional happy effort of nature in a sound system cause an implicit reliance that such efforts will prove successful under every variety of constitutions, or always be adequate to the counteraction of ill directed modes of cure.

To

To a young mind the necessary distinctions which terms are intended to convey are not always present, and first impressions are ever of high importance. The term carious excites instant alarm, and the difference between that state of bone and diseased bone are too often overlooked. That tedious exfoliation, in defiance of the most skilful surgery, will follow denudation of pericranium and periosteum, under some circumstances, is irrefutably true, but it is no less true that apprehension of such an event being unavoidable, has led to practices which have produced the dreaded evil; *e. g.* when exposed bone has assumed an appearance indicative of that destruction having taken place, which sometimes precedes exfoliation, how common has it been to adopt very active measures upon the principle that such an event must necessarily succeed, when an exactly opposite mode of reasoning and of practice would have been nine times out of ten (as observed by Sir William Blizard) successful.

Let not then the young and inexperienced practitioner be needlessly alarmed, when with laudable zeal and anxious thirst after knowledge he reads the above statement, and hastily adopt sentiments which may influence his practice, to his own discredit and the injury of his patient. "Exfoliation," said the sagacious Pott, "from a cranium laid bare by external violence, and to which no other injury has been done than merely stripping it of its covering, is a circumstance which would not so often happen, if it was not taken for granted *that it must be*, and the bone treated according to such expectation."

"Exfoliation is full as often the effect of art as the intention of nature, and produced by a method of dressing, calculated to accomplish such end under a supposition of it being necessary."

A few years ago, a youth of 14 bathed in a canal on a chilly day when much heated; a few hours after he complained of universal pain, had a smart rigor followed by sickness. Continuing thus indisposed for several days I was desired to visit him; he now complained of severe pain near the middle of the left tibia, that all his "other pains had gone there." On examination there was considerable inflammation with some swelling discovered; he was labouring under severe pyrexia with constipation, indicating general and local antiphlogistic treatment, which was immediately instituted and persevered in till it was manifest an abscess in the leg was unavoidable. On this event taking place I was prevented making the necessary opening in due time; the tumour was large and remained in a state fit for the knife several days before it was suffered to be used; upon discharging upwards of a pint of matter, I found found the periosteum denuded to a much greater extent than I could have supposed possible in the time; the subject was become feeble, emaciated, had considerable night sweats with a thin discharge from the wound, gradual thickening and deformity of the limb; a free use of the cinchona and a more generous diet removed these instances of debility, and the sore, in a few weeks, contracted round the denuded bone, which comprehended an extent of near two inches of a very unpleasing aspect. However, as healthy granulation advanced in every direction with great general improvement of the system, I felt encouraged to aim at approximation of the sides of the sore by every possible means position and dressing could suggest. The progress of cure was slow but highly satisfactory; a very small portion of bone ultimately exfoliated, not larger than the size of a little finger nail, when a solid union of parts was effected in a limb which had borne every disheartening appearance that osseous inflammation and great thickening could bestow.

A respectable glazier of this city, middle aged, and long afflicted with dreadful arthritic attacks, was returning from a neighbouring village one tempestuous night, and had the misfortune to be thrown from his horse on a hillock of gravel; pitching upon his head, from which his hat had been previously blown, he received an extensive contusion and detachment of the right side of the scalp. Stunned by the blow he remained on the ground some time, and had barely recovered the possession of his faculties on being brought home an hour afterwards. Upon being called to his assistance I found the integuments so comminuted, swoln, and loaded with gravel, that no idea of replacing the scalp could be entertained. I directed the whole head to be well fomented, and a large emollient cataplasm applied, renewing it every six or eight hours; in a few days free digestion commenced with extensive fœtid discharge; cinchona, wine, and nutritive diet corrected and moderated this state; very considerable sloughing was followed by as healthy granulations as could be expected; the middle of the right parietal bone was bare to the extent of an inch, of nearly a circular shape, it had a faded brown appearance. I was anxious concerning the result, but determined upon a diligent application of adhesive plaister to facilitate the union of the soft parts over it; in the course of a month this was effected, so that the spot now formed a firm shining cicatrix of the size and shape of a small almond.

I am, Gentlemen,

Your obedient Servant,

GEORGE NESSE HILL.

*Chester, March* 21, 1811.

